# A Facile Method to Synthesize 3D Pomegranate-like Polydopamine Microspheres

**DOI:** 10.3389/fbioe.2021.737074

**Published:** 2021-12-21

**Authors:** Farnaz Ghorbani, Behafarid Ghalandari, Chaozong Liu

**Affiliations:** ^1^ Department of Orthopedics, Shanghai Pudong Hospital, Fudan University Pudong Medical Center, Shanghai, China; ^2^ State Key Laboratory of Oncogenes and Related Genes, Institute for Personalized Medicine, School of Biomedical Engineering, Shanghai Jiao Tong University, Shanghai, China; ^3^ Institute of Orthopaedic and Musculoskeletal Science, University College London, Royal National Orthopaedic Hospital, London, United Kingdom

**Keywords:** biomaterials, biomimetic, nanoparticles, microstructure, protein interaction

## Abstract

Nanospheres have found versatile applications in the biomedical field; however, their possible harmful effects on immune and inflammatory systems are also a crucial concern. Inspired by a pomegranate structure, we demonstrated a novel structure for the nanostructured microspheres to overcome the drawbacks of nanospheres without compromising their merits. In this study, 3D pomegranate-like polydopamine microspheres (PDAMS) were synthesized by self-oxidative polymerization of dopamine hydrochloride. Herein, controlling the pH during polymerization led to synthesizing homogeneous agglomerated nano-sized spheres (400–2000 nm) and finally forming tunable and monodisperse micron-sized particles (21 µm) with uniform spherical shape porous microstructure. PDAMS interaction with the potential targets, Bone morphogenetic protein-2 (BMP2), Decorin, and Matrilin-1, was investigated via molecular calculations. Theoretical energy analysis revealed that PDAMS interaction with BMP2, Decorin, and Matrilin-1 is spontaneous, so that a protein layer formation on the PDAMS surface suggests application in bone and cartilage repair. It was also observed that PDAMS presented *in-vitro* degradation within 4 weeks. Here, disappearance of the UV-VIS spectrum peak at 280 nm is accompanied by the degradation of catechol groups. Pomegranate-like PDAMS support the biomimetic formation of hydroxyapatite-like layers, making them appropriate candidates for hard tissue applications. Herein, the appearance of peaks in XRD spectrum at 31.37, 39.57, 45.21, and 50.13° attributed to hydroxyapatite-like layers formation. All these results demonstrated that self-oxidative polymerization under a controllable pH can be a green and straightforward technique for preparing the pomegranate-like PDAMS and providing an innovative basis for further pre-clinical and clinical investigations.

## Introduction

Nanospheres are abundantly used in the biomedical area due to their superior properties. However, the toxicity of the nanoparticles, as well as their possible harmful effects on immune and inflammatory systems, severe aggregation, and high inter-particle resistance, are the main concerns in the biomedical application. In order to overcome these limitations, the authors have developed pomegranate-like spheres inspired by the structure of a pomegranate. *In-vitro* study has demonstrated these biomimetic nanostructured microspheres can overcome the drawbacks of conventional nanoparticles without compromising their merits. Accordingly, such a design can have some benefits as follows: 1) facilitates mass transfer, such as cell nutrients and metabolic waste (Ji et al., 2021); 2) supports adequate swelling with no differences in the final size of microspheres owing to the presence of void space, which can make them suitable structures for the size-dependent applications (Liu et al., 2014); 3) promotes the stability of protein-microsphere interactions (Blekemolen et al., 2018; Luo et al., 2019); 4) provides more anchorage sites and a high level of surface energy to improve cells attachment, facilitate cells migration, proliferation, and differentiation (Zhou et al., 2018); 5) primary agglomerated nanospheres maintain structural integrity and prevent microspheres from crack under the applied stresses (Liu et al., 2014), and 6) improves drug-loading capacity and applies more control on release rate (Ji et al., 2021).

Dopamine, a neurotransmitter, can be spontaneously polymerized to polydopamine (PDA). PDA, a mussel-inspired and melanin-like material, has found expanded application in the biomedical area, including surface modification (Liu et al., 2021), drug delivery (Wang et al., 2021), tissue engineering (bone (Deng et al., 2019), tooth (Hasani-Sadrabadi et al., 2019), nerve (Yan et al., 2020), muscle (Tian et al., 2018), skin (Tang et al., 2019), cartilage (Han et al., 2018), vessel (Zhou et al., 2017)), photothermal therapy (Ruan et al., 2021), microfluidic systems (Kanitthamniyom and Zhang, 2018), and bioimaging (Nurunnabi et al., 2013). Biocompatibility and minimal inflammatory response, high level of hydrophilicity, excellent bioactivity, bio-adhesive properties, anti-bacterial potential, thermal stability, cost-effective and green synthesis technology can be responsible for all-round development (Iqbal et al., 2012; Liu et al., 2013a; Liu et al., 2013b; Gong et al., 2017; Zhao et al., 2019; Steeves and Variola, 2020; De Guzman et al., 2021; Gholami Derami et al., 2021; Park et al., 2021; Sun et al., 2021). According to previous publications, the synthesis of PDA strongly depends on the chemical properties of reaction media; therefore, the concentration, types of alcohol, the ratio of water to alcohol, and pH can affect the polymerization process, as well as the final shape, size, and uniformity of the synthesized structure (Jiang et al., 2014; Ghorbani et al., 2019a). Accordingly, monodisperse 3D pomegranate-like PDA microspheres (PDAMS) were synthesized based on the homogeneous and controlled-agglomeration of PDA nanospheres to compensate for the drawbacks of single nanospheres.

In this work, a green and facile technique was introduced to synthesize the pomegranate-like PDAMS. The resultant PDAMS were subsequently characterized with respect to their morphological property and chemical composition, degradation behavior, and biomineralization potential. In addition, PDAMS interaction with the potential targets, bone morphogenetic protein-2 (BMP2), Decorin, and Matrilin-1, was investigated via molecular calculations, which shows their suitability for hard tissue applications.

## Materials and Methodology

Nanostructured-PDAMS were synthesized by the oxidative polymerization of dopa-HCl in pH-controlled conditions. Briefly, a solution of isopropanol-DI-water (with a volume ratio of 5:2) was prepared, and the pH was adjusted to 8.5 by adding Tris-buffer (10 mmol/L). Then, dopa-HCl with a concentration of 1.5 mg/ml was added to the prepared solution dropwise. The mixture was stirred at 250 rpm for 3 days at ambient temperature. The pH of the solution was monitored every 5 h and was adjusted to 8.5 by the addition of ammonia solution. Finally, the solution was centrifuged with DI-water, and sediments were lyophilized.

The microstructure of the nanostructured-PDAMS was examined using a field-emission scanning electron microscopy (FE-SEM, MIRA3, TESCAN Co., Czech Republic) at an accelerating voltage of 15 kV after coating a thin layer of gold to reduce charge density. The size distribution was analysed by Image measurement software (KLONK Image Measurement Light, Edition 11.2.0.0). The polymerization was evaluated using Fourier-transforminfrared spectroscopy and proton nuclear magnetic resonance (H-NMR, ECX 400, JEOL Co., United States) spectroscopy. H-NMR spectroscopy was performed through dissolving the microspheres in deuterium oxide (D_2_O): DMSO-d_6_ (1: 1). The spectra were recorded by a 4 mm MAS probe under an applied frequency of 5,000 Hz at 303 K. *In-vitro* degradation of spheres was evaluated by FE-SEM micrographs and ultraviolet-visible absorption (UV-VIS, Lambd25, Perkin-Elmer Co., United States) spectroscopy. The nanostructured-microspheres were immersed in PBS solution at 37 ± 0.5 °C for 4 weeks before use. At the end of each week, the PBS solution was refreshed. Finally, the samples were washed with DI-water and were lyophilized. The *in-vitro* bioactivity of microspheres was determined by immersion of microspheres in the SBF solution at 37 ± 0.5°C for 4 weeks. The SBF solution was refreshed every 2 days. Finally, the samples were washed with DI-water and were lyophilized. The mineralized microspheres were characterized using FE-SEM micrographs and phase analysis by X-ray diffraction (XRD, PW3710, Philips Co., Netherlands) using Cu-Kα radiation under the operating conditions of 40 kV and 30 mA. XRD patterns compared with JCPDS standards.

Molecular docking calculation using AutoDock Vina (Trott et al., 2009) was performed to evaluate PDAMS binding potential to the critical target proteins of Decorin, Matrilin-1, and BMP2. The 3D structures of BMP2 (PDB ID: 1REW (Keller et al., 2004)), Decorin (PDB ID: 1XCD (Scott et al., 2004)) and Matrilin-1 (PDB ID: 1AQ5 (Dames et al., 1998)) were obtained from the RCSB Protein Data Bank. Docking calculations were carried out with the basic unit size of PDAMS (<2 nm) as the repeated-basic unit, which expanded the computations to acquire the full-length scale. The 3D structure of the repeated-basic unit of PDAMS was modeled with the Hyperchem program, and its geometry was optimized by minimizing the energy using the B3LYP keyword with the 6-31G (d, p) basis set implemented in Gaussian 98 package. The molecular docking simulations were performed according to the classical preparation instruction (Abbasi-Tajarag et al., 2016; Gholamian et al., 2017; Shafaei et al., 2017). The VMD package (Morris et al., 2009) and the AutoDock Tools 1.5.4 (Humphrey et al., 1996) were used for input file preparation and data analysis.

## Results and Discussion

**FIGURE 1:**
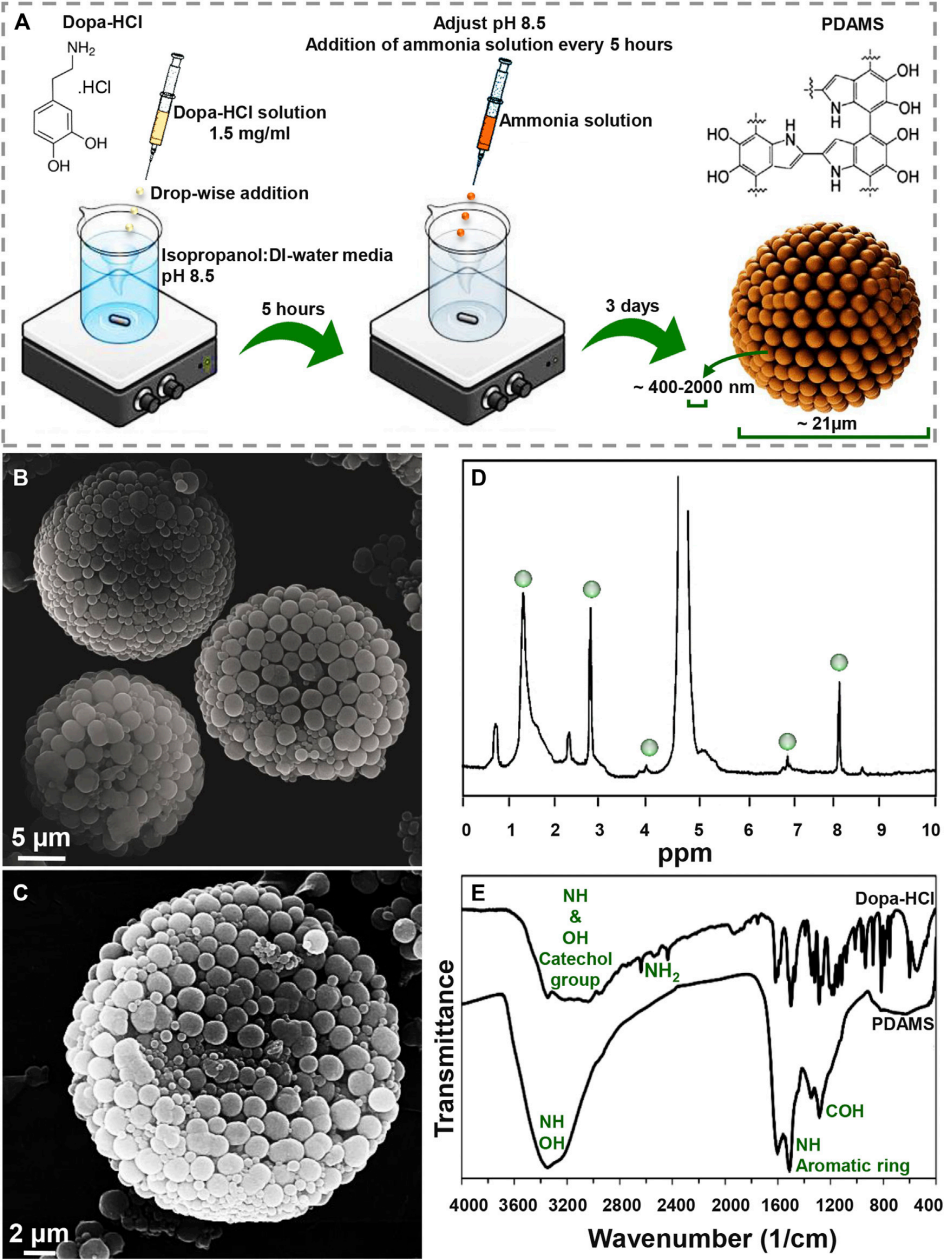
Morphological and chemical observation of pomegranate-like PDAMS **(A)** A schematic of the synthesis process **(B)** FE-SEM micrographs of homogeneous nanostructured PDAMS, which were synthesized by spontaneous oxidation under controlled pH **(C)** FE-SEM micrographs of the inner structure of PDAMS **(D)** H-NMR and **(E)** FTIR of polymerized microspheres.

Figure 1A presents a schematic of the synthesis procedure of pomegranate-like PDAMS. In this study, the particles were synthesized under self-oxidative polymerization of dopa-HCl in pH 8.5, which was then controlled to be stable within 3 days of synthesis. According to FE-SEM image (Figure 1B, C), tunable, spherical, and 3D pomegranate-like architecture PDA particles were prepared with an average size distribution of 21.92 ± 2.07 µm and smooth surface. Homogeneous and uniform agglomeration of primary nano-sized PDA spheres (ranging from 400 nm to 2 µm) led to the formation of the nanostructured-PDAMS with interconnected porous-like structures. This phenomenon may be attributed to controlled-pH, while in a similar study (Ghorbani et al., 2019b) there is no sign of homogeneous agglomeration of PDA nanospheres. Moreover, the void space between the agglomerated nanospheres (ranging from 20–300 nm) led to the formation of a 3D pomegranate-like structure that can facilitate and promote protein-microsphere interactions (Blekemolen et al., 2018; Luo et al., 2019).

The H-NMR spectra of PDAMS (Figure 1D) determined the aliphatic protons at 1.4, 2.9, and 4 ppm, which are attributed to (CH_2_−C), (CH_2_−C/CH_2_−N), and (CH_2_−N), respectively. The observed peaks at 7 and 8.1 ppm could be assigned to aromatic CH in indole. FTIR spectra (Figure 1E) demonstrated aromatic OH stretching vibration at 3,143, 3,065, 3,034, and 2,957 cm^−1^ in dopa-HCl chemical composition. Additionally, intermolecular hydrogen bonds were detected with a broad peak at 3,000–3,400 cm^−1^. Moreover, disappearance of NH_2_ characteristic peaks in PDMS compared with dopa-HCl, which can be observed at 3,210, 2,745, 2,640, and 2,435 cm^−1^, confirmed the polymerization process. Besides, the vibration of phenolic OH and NH in the catechol groups was proved by the peak that was observed at 3,200–3,500 cm^−1^. In addition, the presence of the aromatic ring and NH vibration was determined with a sharp peak at 1,605 cm^−1^. Moreover, both NH and CO vibrations were identified with the absorption peaks at 1,510 and 1,120 cm^−1^, respectively. The characteristic peaks at 1,345 and 1,284 cm^−1^ were assigned to phenolic OH.

The pH of reaction media has been known as the influencing factor on PDA formation. Generally, the alkali media oxidizes dopa-HCl followed by deprotonation of amine groups under 1,4- Michael addition reaction. Leucodopaminechrome, the product of the reaction, is oxidized and then rearranged to 5,6-dihydroxyindole. The interaction between o-quinone in 5,6-dihydroxyindole structure and catechol groups leads to the formation of PDAMS (Yan et al., 2013; Xiong et al., 2014). Moreover, polymerization of dopa.HCl can lead to pH reduction due to the ring-closing reaction of the monomer and the elimination of hydrogen atoms in the monomer chemical structure (Ju et al., 2011). So, the formation of homogeneous nanostructured PDAMS can be attributed to pH control during the polymerization process. The stability of the pH on higher values can be attributed to the neutralization of acidic monomer, long-lasting, and easy deporatonation of catechol groups, as well as the formation of the nanostructured-microspheres. Also, by keeping the pH in stable alkali values, complete spontaneous oxidation of dopamine will happen at a slower rate. When reactions of dopaminechrome to 5,6-dihydroxyindole and indolequinone are slow, it leads to accumulation reaction products in the media (Salomäki et al., 2018), which accompanies intra/intermolecular cross-linking reactions strong π-π interaction (Ju et al., 2011) that lead to the formation of pomegranate-like PDAMS.

The ratio of isopropanol to water (5:2) was determined in terms of the Hansen solubility parameters theory, as indicated in Eqs 1, 2 (Jiang et al., 2014). Where D, P, and H are dispersive, polar, and hydrogen bonding solubility parameters, respectively. R_a_ and Ф are the levels of conformity and the volume fraction of each composition, respectively. Eq. 1 * 2 indicate that the higher degree of uniformity was originated from a high degree of solubility (Lower R_a_ value).

$${\text{R}}_{\text{a}}=1\text{/}2{\left[4{\left({\text{D}}_{\text{solv}}{-\text{D}}_{\text{solu}}\right)}^{2}+{\left({\text{P}}_{\text{solv}}{-\text{P}}_{\text{solu}}\right)}^{2}+{\left({\text{H}}_{\text{solv}}{-\text{H}}_{\text{solu}}\right)}^{2}\right]}^{\text{0}\text{.}5}$$ (1)

$$\text{D}{\left(\text{P,H}\right)}_{\text{blend}}=\Sigma \Phi_{n.comp}\text{D}{\left(\text{P,H}\right)}_{n.comp}$$ (2)

**FIGURE 2:**
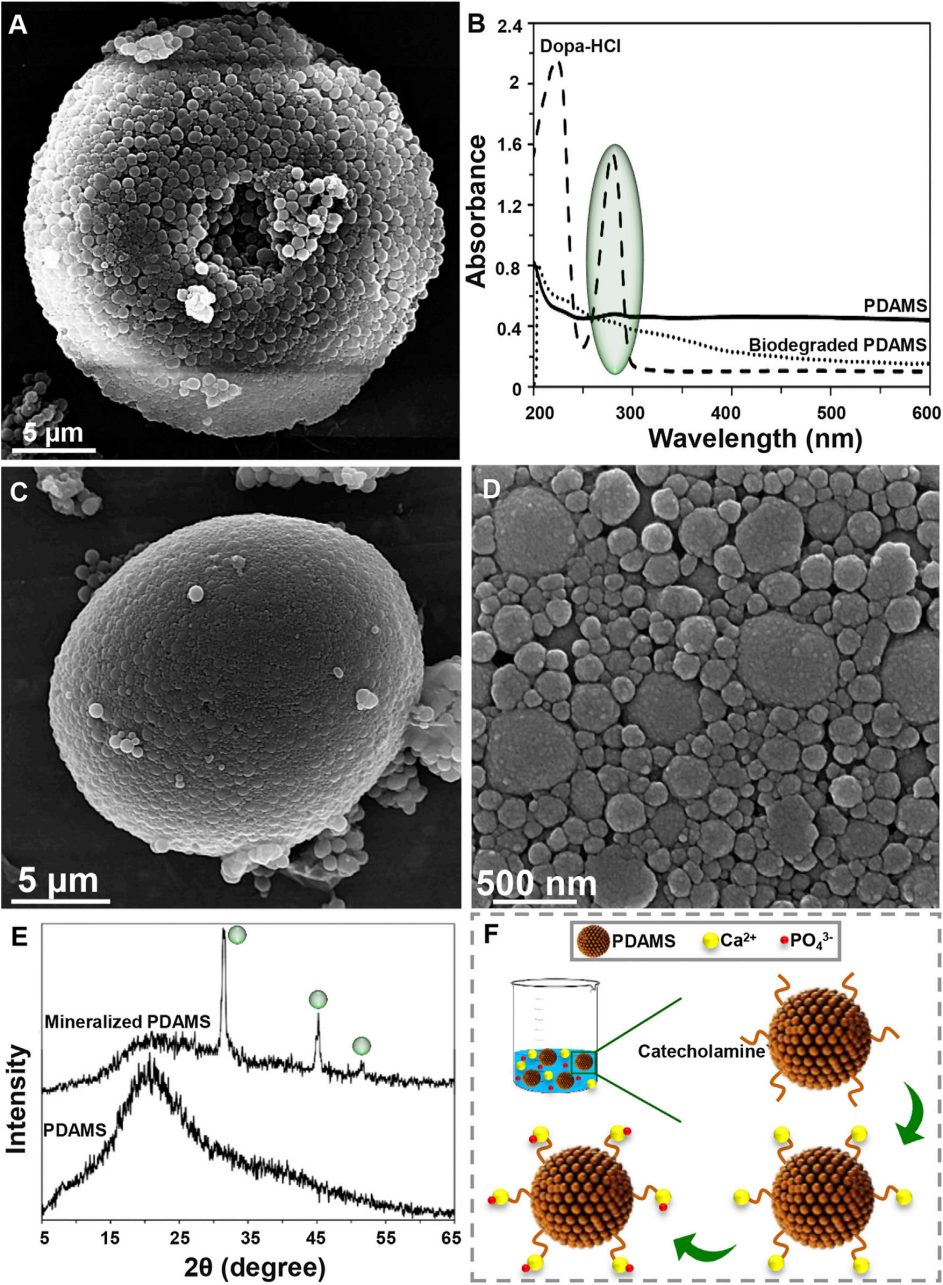
Degradation and bioactivity of pomegranate-like PDAMS **(A)** FE-SEM image and **(B)** UV-VIS spectra of *In-vitro* degraded PDAMS after a 4-weeks immersion in the PBS solution **(C, D)** FE-SEM image and **(E)** XRD spectra of *In-vitro* biomimetic formation of hydroxyapatite-like layers on the PDAMS after a 4-weeks immersion in the SBF solution **(F)** Schematic of biomineralization of hydroxyapatite-like layers on PDAMS.

Degradability of the synthesized structures is an essential factor in the biomedical area that affects the size, shape, and chemical composition of particles (Han et al., 2018). Degradation of PDAMS was illustrated in Figure 2A, B. Based on the FE-SEM micrographs (Figure 2A), a 4-week immersion of the bioinspired-microspheres in the PBS solution slightly altered the surface, while a low degree of deformation was observed, indicating the stability of PDAMS. According to UV-VIS spectra (Figure 2B), the polymerization of dopa-HCl has led to the reduced intensity of the catechol groups peak at 280 nm, which was strongly affected by the pH of reaction media. However, the immersion of PDAMS in the PBS solution is shown to be accompanied by the degradation of catechol groups and disappearance of the related peak at 280 nm. Since catecholamine groups in the chemical composition of PDAMS can facilitate the absorption of the PBS solution and make degradation possible, so degradation of this hydrophilic functional group may reduce the degradation speed. Notably, the size of microspheres was known as an active factor in the degradation process. The microspheres with lower size distribution (less than 300 µm) have a higher tendency to homogeneous degradation (Anderson and Shive, 2012). However, microspheres with a size distribution of more than 10 µm are considered large for the phagocytosis process. Therefore, homogeneous degradation terminates to the reduced size and also facilitates phagocytosis by foreign body giant cells and macrophages (Tabata and Ikada, 2005). Additionally, void space may affect degradation. It is expected that PDAMS provides a higher permeation to fluids and accelerates degradation than dense structures (Anderson and Shive, 2012).

Potential of particles to support the biomineralization of hydroxyapatite-like layers can make them suitable for hard tissue repair. Also, biomimetic hydroxyapatite has found versatile application due to lack of immune rejection, trauma, and limited supply, among other factors exhibited in other hydroxyapatite sources (Li et al., 2008; Ghorbani et al., 2020). Bioactivity of the nanostructured-PDAMS after immersion of particles in the SBF solution for 4 weeks was shown in Figure 2C–F. From the FE-SEM examinations, it was observed that a layer of calcium phosphates with a nanoscale structure has been homogeneously precipitated on the surface of microspheres. The biomimetic mineralization of hydroxyapatite was confirmed by the XRD analysis. According to XRD spectra, the immersion of microspheres in the SBF solution led to the absorption of minerals. The appearance of peaks at 31.37, 39.57, 45.21, and 50.13° attributed to the reflections of (211), (130), (222), and (231) crystalline plates, respectively, which was accompanied with reduction in intensity of the amorphous peak of PDAMS at 2θ angles of approximately 20°. Figure 2F presents a schematic of biomineralization. When the microspheres immerse in the SBF solution, PDAMS releases H^+^ into the media. Then, negatively surface charge and free catecholamine moieties tend to absorb calcium ions from media and reduce the system’s energy. Therefore, the deposited calcium changes the surface charge and provides a suitable site for absorbing phosphate ions and forming the hydroxyapatite layer (Ding et al., 2016). Also, void space in pomegranate-like PDAMS provides a higher surface area to facilitate the absorption and diffusion of body fluids (Liu et al., 2014) to form biomimetic hydroxyapatite.

**FIGURE 3:**
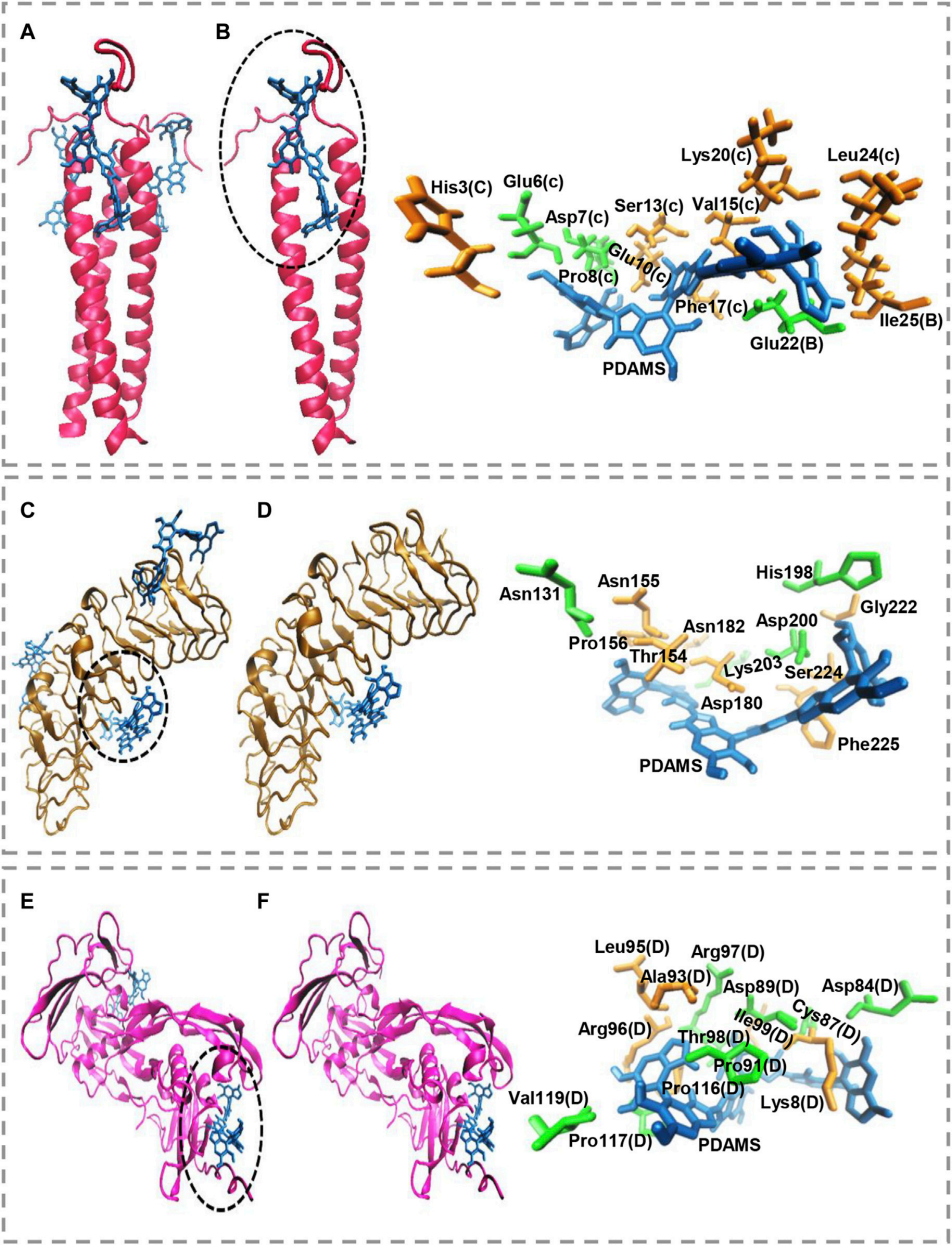
Molecular docking simulation of PDAMS binding to target proteins. The PDAMS is shown as licorice in blue as well as Matrilin-1, Decorin, and BMP2 are shown as cartoon in red, ochre, and magenta, respectively. The main driving forces to form the target proteins-PDAMS complex are Van der Waals interaction and hydrogen bond. Van der Waals interaction and hydrogen bond are shown in orange and green, respectively **(A)** Matrilin-1 interaction with PDAMS **(B)** PDAMS is located between two α-helixes of Matrilin-1 at the head of the coiled-coil. The best mode of PDAMS interaction with Matrilin-1 in the binding site **(C)** Decorin interaction with PDAMS **(D)** The best mode of PDAMS binding to Decorin and the specific interactions in the binding site **(E)** BMP2 interaction with PDAMS **(F)** The best mode of PDAMS interaction with BMP2 and the specific interactions in the binding site.

Molecular docking calculations show the surface of the target proteins structurally adapt to PDAMS’s surface (Figure 3). In other words, considering the real size of the PDAMS, there is a structural complementary between the surface of the PDAMS and the target proteins. As shown in Figure 3A, Matrilin-1 binds to PDAMS from the head of the α-helical coiled-coil. Docking calculations show the PDAMS are located between two α-helixes (Figure 3B). The computations indicate the identical behavior of each two α-helixes interaction with PDAMS since Matrilin-1 is a homotrimer. The best mode of PDAMS interaction with Matrilin-1 considering the pose clustering of docking calculations is shown in Figure 3B. Docking analysis demonstrates that van der Waals interaction and hydrogen bond are the main driving forces for Matrilin-1 binding to the PDAMS surface.

As shown in Figure 3C, PDAMS is located on the surface of the Decorin. Molecular docking calculations demonstrate the entire Decorin surface is prone to interact with the PDAMS. The best pose clustering of docking simulations is considered to show the details of Decorin interaction with PDAMS (Figure 3D). Docking analysis reveals the van der Waals interaction and hydrogen bond are the main driving forces for Decorin binding to the PDAMS surface. BMP2 interaction with PDAMS calculated by docking simulation is also shown in Figure 3E. Docking calculations illustrate the lateral surfaces of BMP2 interact with PDAMS. The best pose clustering of docking calculations is considered to show the details of BMP2 interaction with PDAMS (Figure 3F). Docking analysis indicates the van der Waals interaction and hydrogen bond are the main driving forces for BMP2 binding to the PDAMS surface.

PDAMS’s chemical structure and surface property are the main reasons for the predominance of obtained driving forces in the complex formation between PDAMS and target proteins. Also, the theoretical binding energy for all proteins indicates the binding process is spontaneous. The obtained value for PDAMS interaction with Matrilin-1, Decorin, and BMP2 is -39.71 kJ mol^−1^, -40.96 kJ mol^−1^, and -45.98 kJ mol^−1^, respectively. Thus, docking simulations demonstrate PDAMS has a greater affinity to the bone protein than the cartilage protein. Hence, it suggests that more bone protein attaches to the surface of PDAMS. However, altogether, there is desirable structural compatibility between target proteins and PDAMS. Therefore, the structural adaptation will lead to forming a layer of proteins on the PDAMS surface, which theoretically suggested boosting the application of PDAMS in bone and cartilage repair.

## Conclusion

In this study, a facile method was applied to synthesize 3D pomegranate-like PDAMS. The nanostructured microspheres were synthesized with self-oxidative polymerization of dopa-HCl in alkali media. In this method, the pH was controlled and fixed to 8.5 during the polymerization process that was led to the homogeneous agglomeration of nano-sized spheres and formation of tunable and monodisperse micron-sized particles with uniform spherical shape and porous microstructure. The bioinspired-microspheres showed *in-vitro* degradation within 4 weeks; in addition, these microspheres provide active sites for nucleation of hydroxyapatite-like layers. Molecular docking calculations indicate that target proteins have structural complementary with PDAMS and make complex through a spontaneous process that suggests boosting the application of PDAMS in bone and cartilage repair. This work demonstrated that pomegranate-like PDAMS offer a substrate with initial physicochemical and *in-vitro* properties and facilitate further pre-clinical and clinical investigations for hard tissue repair.

## Data Availability

The original contributions presented in the study are included in the article/supplementary material, further inquiries can be directed to the corresponding author.
